# A Compact Four-Port MIMO Antenna for UWB Applications

**DOI:** 10.3390/s22155788

**Published:** 2022-08-03

**Authors:** Aiting Wu, Mingyang Zhao, Pengquan Zhang, Zhonghai Zhang

**Affiliations:** School of Electronics and Information, Hangzhou Dianzi University, Hangzhou 310018, China; wuaiting@hdu.edu.cn (A.W.); my1227@hdu.edu.cn (M.Z.); zhangzhonghai@hdu.edu.cn (Z.Z.)

**Keywords:** UWB antenna, MIMO antenna, compact antenna, polarization diversity, high isolation

## Abstract

A compact four-port multiple-input multiple-output (MIMO) antenna for ultrawideband (UWB) applications is presented in this paper. The proposed antenna has four unit cell antennas. Each unit cell is placed orthogonal to its adjacent elements. The radiation element of each unit cell is composed of a cut semicircular patch and a stepped microstrip feed line. The whole ground on the back side consists of four parts of defective ground and their extended branches, which are connected through a “卍” structure. The main decoupling technology used in the MIMO antenna is polarization diversity. In addition, protruded ground and parasitic elements are added to achieve a higher isolation. This compact antenna has a small area of 45 mm × 45 mm and is printed on a single layer substrate (FR4) with an ε_r_ = 4.4 and a thickness of 1.6 mm. This antenna has an impedance bandwidth (S11 < −10 dB) of 3.1–13.1 GHz (123%) and an isolation of less than −17 dB. The envelope correction coefficient (ECC) is less than 0.02 and the average gain is 4 dBi. The ultrawide bandwidth and compact size of the proposed antenna make it a promising candidate for UWB applications.

## 1. Introduction

UWB technology has become a hot topic in recent research. It is a communication method implemented by a series of pulses with very short period; it is also called pulse communication technology. Compared with traditional narrowband technology, it has many advantages, such as high data rate wireless transmission, rich multipath diversity, and very low power consumption. Due to the overlap between UWB signal frequency range and the existing narrowband signal frequency range, the FCC limits the transmission power of UWB (3.1–10.6 GHz). In order to obtain better performance with the limited transmission power, extensive research has been conducted and many solutions proposed. A promising method is to combine MIMO and UWB technology.

Multiple antennas at the transmitter and receiver are used in MIMO technology. This manages to suppress channel fading due to its multipath characteristics and can significantly improve the spectrum utilization. In order to integrate multiple antenna elements on a small substrate, appropriate decoupling is introduced between antenna elements to increase the isolation.

Many decoupling methods have been proposed. In [1], a neutralization line is added between two radiation elements, and it connects to the elements. The neutralization line contains two metal strips connected via rhombus plate. The line effectively reduces the coupling current at ground and achieves a wideband decoupling current. A high isolation is achieved by exploiting the polarization of the multiple elements [2]. A UWB MIMO antenna with a high isolation (less than −22 dB) by loading a parasitic unit decoupling structure on the floor was proposed in [3]. The parasitic element consists of a T-junction and a pair of symmetrical bending lines. Other decoupling methods include defective ground structure (DGS) [4], protruded ground [5], self-decoupling, electromagnetic band gap (EBG) structures [6], decoupling network, and metamaterial [7]. The frequency selective surfaces (FSSs) [8,9] could enhance the antenna’s gain for UWB frequencies.

In [2], a four-element MIMO antenna with polarization diversity technology has a high isolation and a small size. However, the substrate of this antenna is Rogers TMM4. The material is very expensive. The size of the four-element MIMO antenna mentioned in [10] is small, but the isolation is not good, only less than −16 dB. For the antennas described in [11,12], due to their single decoupling structure, their isolation is not ideal; it is about −15 dB for both.

A compact four-port MIMO antenna for UWB applications with a high isolation and a wide bandwidth is proposed in this paper. The antenna achieves a high isolation, less than −17 dB, by using the polarization diversity method, protruded ground structures, and parasitic elements. Besides, this antenna provides a good impedance matching from 3.1 to 13.1 GHz and has a good average gain of about 4 dBi. In addition, the ECC result is less than 0.02. It indicates that the antenna meets the polarization diversity requirements. In summary, the highlights of the proposed antenna are its ultrawide bandwidth and compact size. At the same time, the other properties are also good, including the isolation, the gain, and the ECC. 

The rest of the paper is organized in the following sections: Section 2 presents the geometry of the antenna. To improve the isolation, three configurations (polarization diversity, protruded ground, and parasitic elements) are analyzed in detail. The wide bandwidth is developed by cutting the current path of the patch. Section 3 presents the measured and the simulated results, including return loss, isolation, ECC, diversity gain (DG), radiation pattern, and gain, with detailed analysis. Section 4 is the conclusion.

## 2. Antenna Design

**Figure 1:**
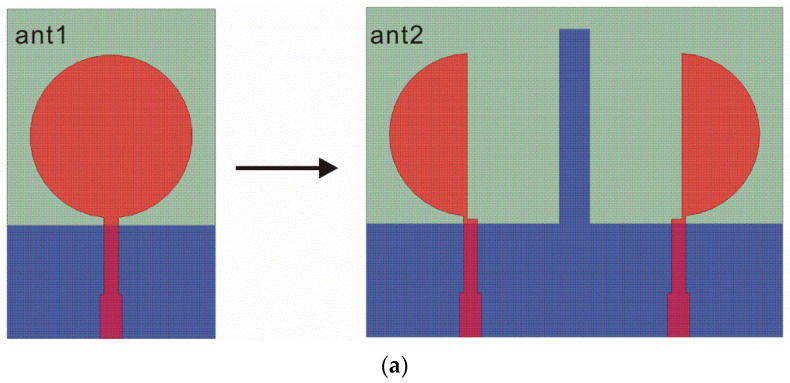

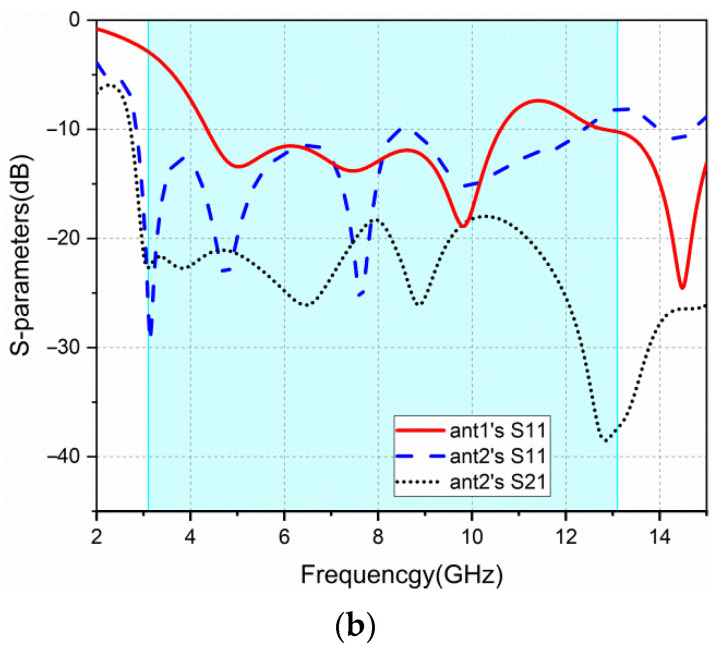
ant1 and ant2: (**a**) geometry; (**b**) S-parameters.

The design of the proposed antenna starts from a circular monopole antenna called ant1, as shown in Figure 1a. The return loss (S11) of ant1 is shown in Figure 1b. The monopole antenna is cut into two identical parts to obtain two semicircular radiation elements for miniaturizing called ant2, as shown in Figure 1a. In order to reduce the mutual coupling between these two elements, protruded ground structure is added. It can be seen from Figure 1b that the ant2′s return loss is improved compared with ant1.

**Figure 2:**
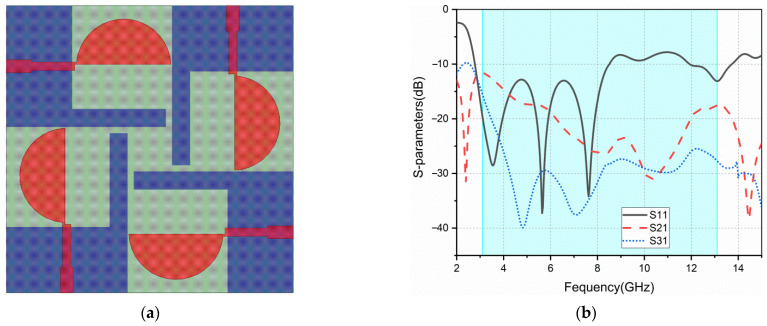
ant3: (**a**) geometry; (**b**) S-parameters.

Based on ant2, a four-element MIMO antenna is constructed, named ant3. It consists of an orthogonal structure that aims to improve the isolation between elements, as shown in Figure 2a. It also retains the protruded ground structure in ant2. The return loss and isolation (S21, S31) of ant3 are shown in Figure 2b. The S11 of ant3 is poor in high-frequency band, and the isolation of this antenna is above −15 dB in the low-frequency band.

**Figure 3:**
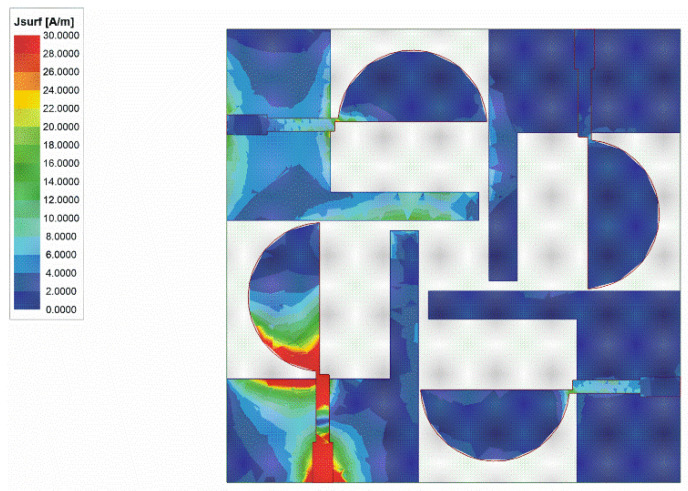
Surface current distribution at 6.5 GHz for ant3.

**Figure 4:**
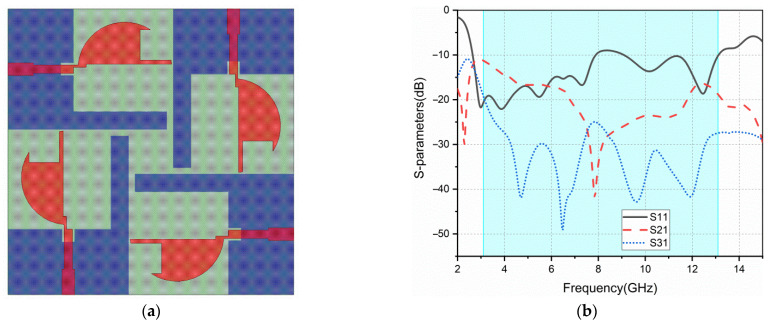
ant4: (**a**) geometry; (**b**) S-parameters.

Figure 3 depicts the surface current distribution at 6.5 GHz of ant3. In order to improve the bandwidth, the geometry of the antenna patch is changed to adjust the current distribution, trying to improve impedance matching. As shown in Figure 4a, the result after cutting the patch of ant3 is ant4. Figure 4b shows the return loss and isolation of ant4. Both the bandwidth and isolation have been improved. However, the isolation still does not meet the requirement in some frequency bands.

**Figure 5:**
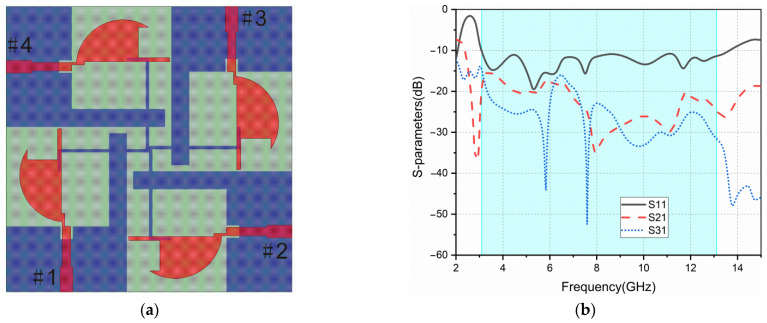
ant5: (**a**) geometry; (**b**) S-parameters.

To further improve the isolation, a “卍” structure is introduced on the metal ground side, as shown in Figure 5a. This generates ant5. It can be seen in Figure 5b, after adding this structure, that the isolation between adjacent elements is significantly improved. This is because the magnitude of mutual coupling currents generated between adjacent elements is the same, while their directions are opposite. They offset each other after passing through the “卍” structure. Besides, the structure also improves the impedance matching of the antenna, and produces a better return loss.

**Figure 6:**
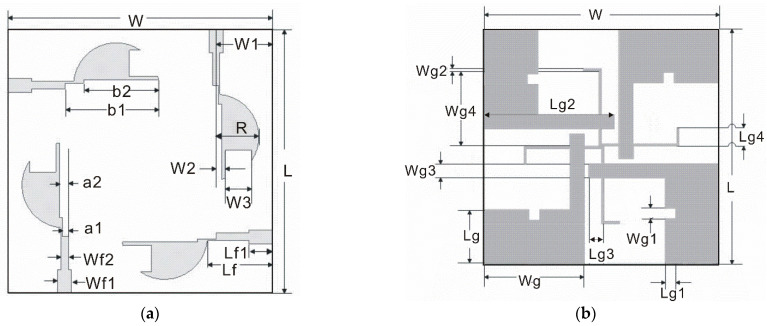
The schematic diagram of the proposed antenna: (**a**) top; (**b**) bottom.

The schematic diagram of the final proposed antenna (ant5) is shown in Figure 6. The antenna’s geometric parameters are listed in Table 1.

## 3. Results and Discussion

**Figure 7:**
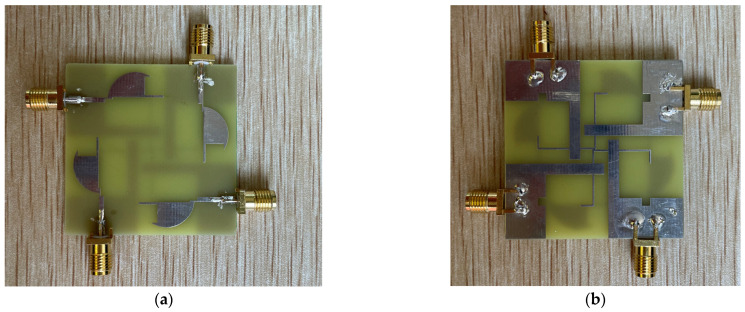
Photograph of the fabricated antenna: (**a**) top; (**b**) bottom.

The proposed antenna is fabricated on a FR4 substrate with a thickness of 1.6 mm, as shown in Figure 7. The measured results are presented and discussed in the following subsections.

### 3.1. S-Parameters

**Figure 8:**
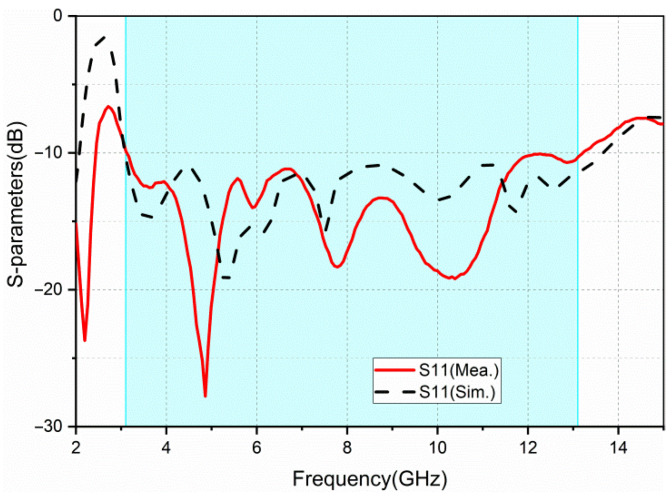
Simulated and measured return loss (S11).

The simulated and measured return loss results of port 1 are provided in Figure 8. The simulated bandwidth (S11 < −10 dB) is 10.5 GHz (3.1–13.6 GHz) and the measured bandwidth is 10 GHz (3.1–13.1 GHz). Due to the manufacturing process, welding problems, and substrate quality, there are slight discrepancies between the simulated and the measured results.

**Figure 9:**
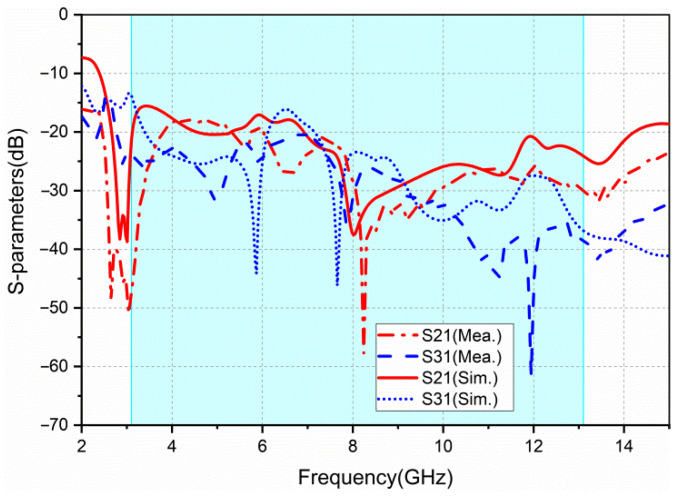
Simulated and measured isolation (S21, S31).

The simulated and measured isolation results between port 1 and port 2, and between port 1 and port 3, are given in Figure 9. The isolation of adjacent elements is less than −17 dB, and that of diagonal elements is less than −20 dB. Because the antenna is completely symmetric, these two parameters only are enough to characterize the antenna’s isolation. 

**Figure 10:**
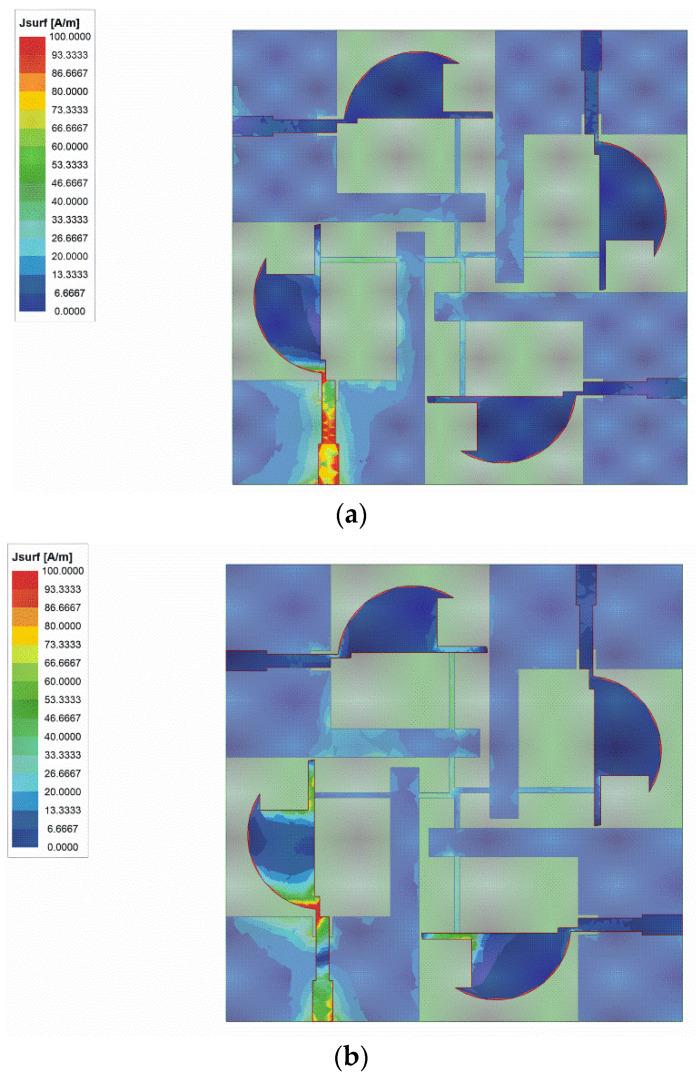

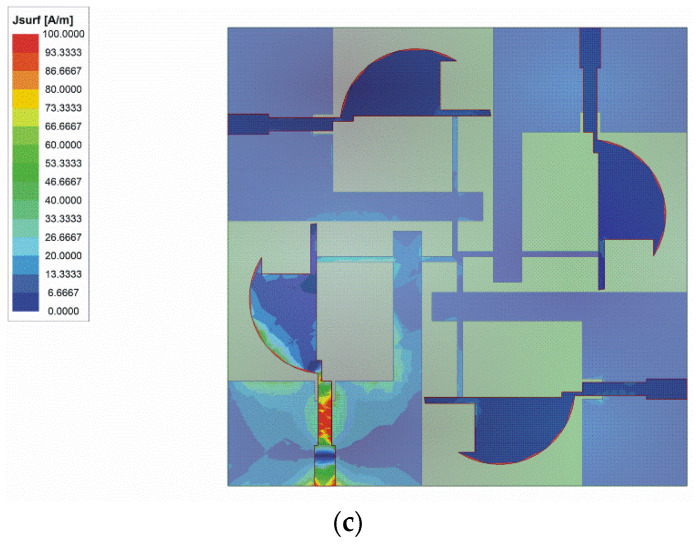
The surface current distribution at: (**a**) 3.5 GHz; (**b**) 7 GHz; (**c**) 10 GHz.

The surface current distribution of the antenna is shown in Figure 10. All the ports of the prototype are terminated to 50 Ω broadband matched loads, except the excited one. The solution frequencies are 3.5 GHz, 7 GHz, and 10 GHz, respectively. As shown in Figure 10, the currents are confined within the area of the active element, protruded ground, and parasitic elements. In the adjacent ports, the currents are very low. The surface current distribution exhibits good isolation.

### 3.2. Diversity Performance

The diversity performance of the UWB-MIMO antenna system is evaluated by ECC, diversity gain (DG), and total active reflection coefficient (TARC).

ECC parameters reflect the degree of correlation between the adjacent elements of MIMO antennas. For a MIMO system, the ECC parameters are expected to be less than 0.5. When the radiation efficiency is high, ECC could be calculated from the following equation [13,14]:

(1) $$ECC=\frac{{\left|S_{ii}^{*}S_{ij}+S_{ji}^{*}S_{jj}\right|}^{2}}{\left(1-{\left|S_{ii}\right|}^{2}-{\left|S_{ji}\right|}^{2}\right)\left(1-{\left|S_{jj}\right|}^{2}-{\left|S_{ij}\right|}^{2}\right)}$$

**Figure 11:**
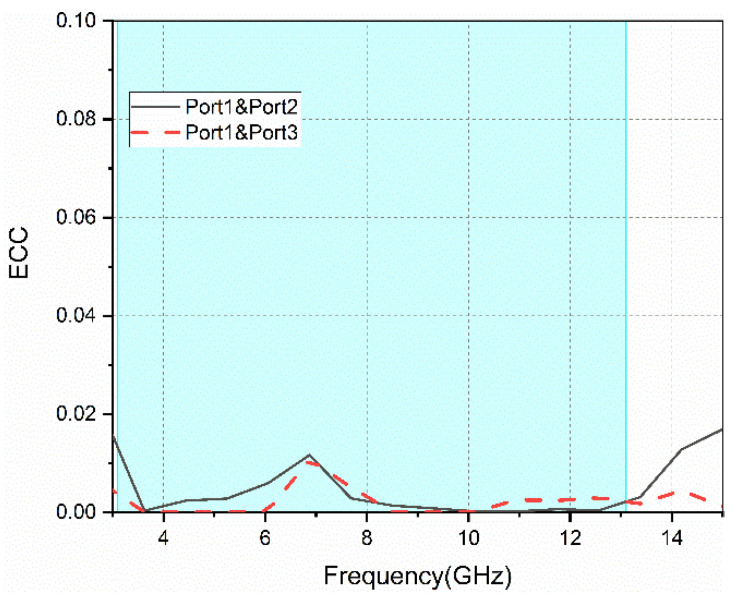
The ECC parameters between different ports.

The ECC parameters between the antenna’s ports are shown in Figure 11; these are less than 0.02 in the operating frequency band. This indicates good isolation between ports.

DG evaluates the quantified improvement in signal-to-noise ratio when the antennas in the MIMO system receive the RF signal. Equation (2) provides the diversity gain [15].

(2) $$DG=10\sqrt{1-{\left|ECC\right|}^{2}}$$

**Figure 12:**
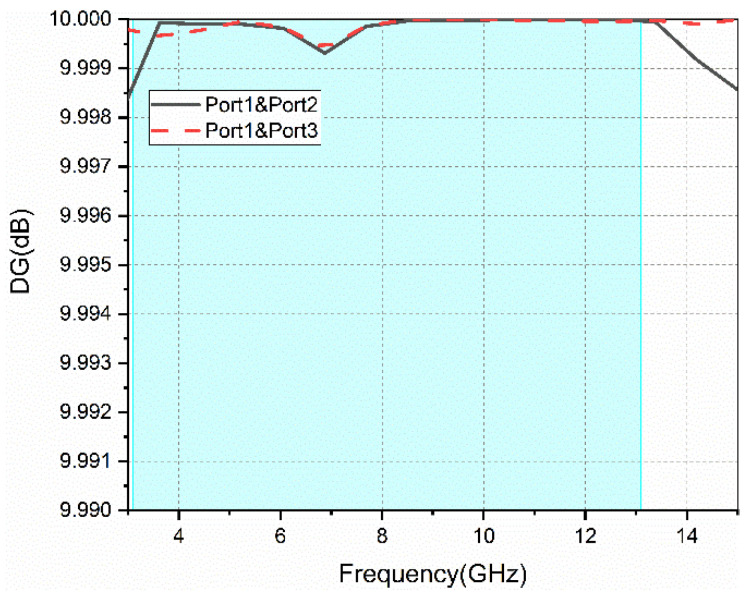
Diversity gain between different ports.

The diversity gain of the proposed antenna can be calculated by the ECC. The DG parameters between different ports are given in Figure 12. A larger value of DG indicates better diversity characteristics. Figure 12 shows that over the whole operating frequency band (3.1–13.1 GHz), the DG values are above 9.9985.

TARC is defined as the ratio of the square root of the total reflected power to the square root of total incident power. It could accurately characterize the effective operating bandwidth of the whole MIMO antenna system and the effect of the change in the phase of signal on the bandwidth of the MIMO antenna. Equation (3) can be used to calculate TARC of the four-port MIMO antenna [13].

(3) $$TARC=\frac{\sqrt{\begin{array}{c} {\left|\left(S_{11}+S_{12}e^{j\theta }+S_{13}e^{j\theta^{\prime }}+S_{14}e^{j\theta^{\prime \prime }}\right)\right|}^{2}+{\left|\left(S_{21}+S_{22}e^{j\theta }+S_{23}e^{j\theta^{\prime }}+S_{24}e^{j\theta^{\prime \prime }}\right)\right|}^{2}\\ +{\left|\left(S_{31}+S_{32}e^{j\theta }+S_{33}e^{j\theta^{\prime }}+S_{34}e^{j\theta^{\prime \prime }}\right)\right|}^{2}+{\left|\left(S_{41}+S_{42}e^{j\theta }+S_{43}e^{j\theta^{\prime }}+S_{44}e^{j\theta^{\prime \prime }}\right)\right|}^{2} \end{array}}}{2}$$

**Figure 13:**
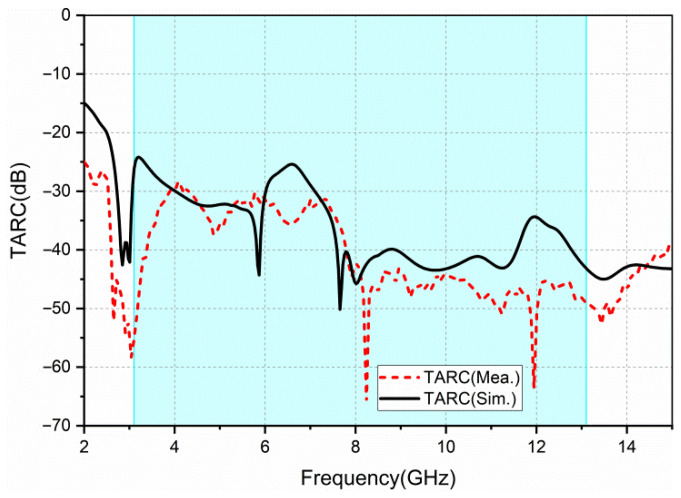
Simulated and measured TARC.

We obtain the average TARC curve by selecting 10 sets of random phases (θ, θ’, θ’’) as shown in Figure 13. TARC typically takes less than 0 dB to characterize the antenna’s performance. The measured TARC obtained is less than −25 dB. It shows that the proposed antenna has a good performance in the MIMO system.

### 3.3. Radiation Pattern, Gain, and Efficiency

**Figure 14:**
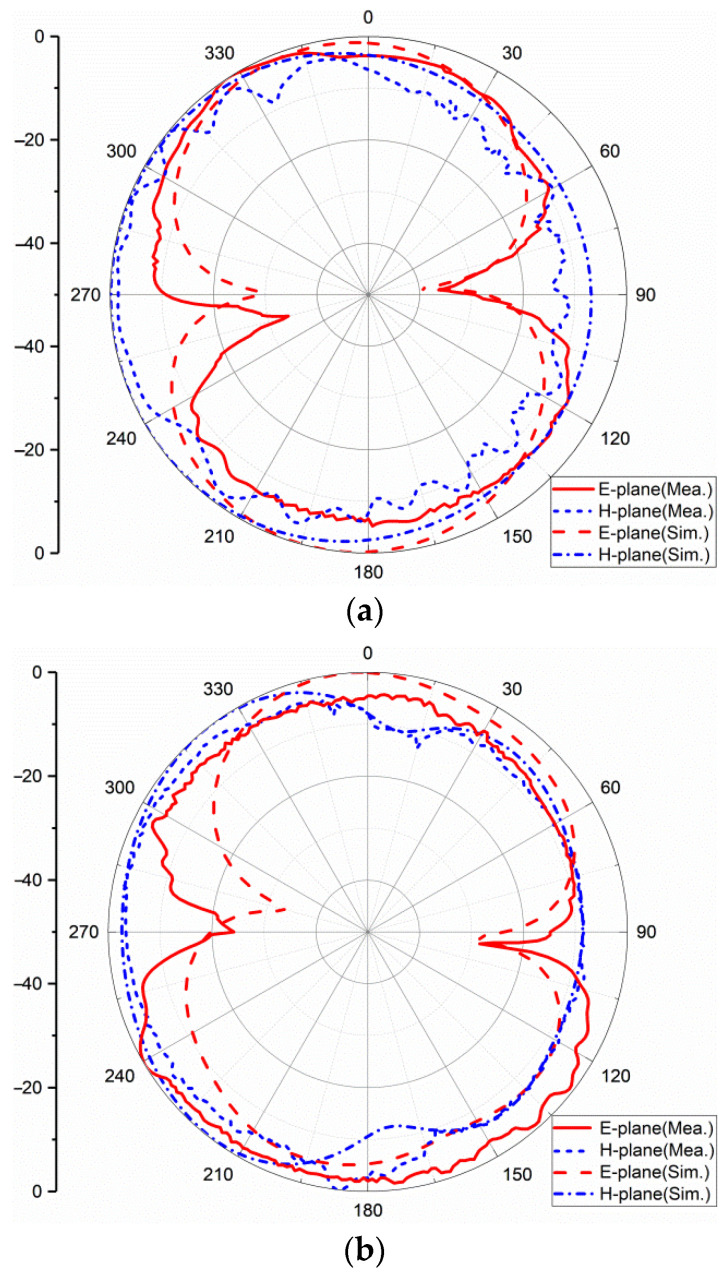

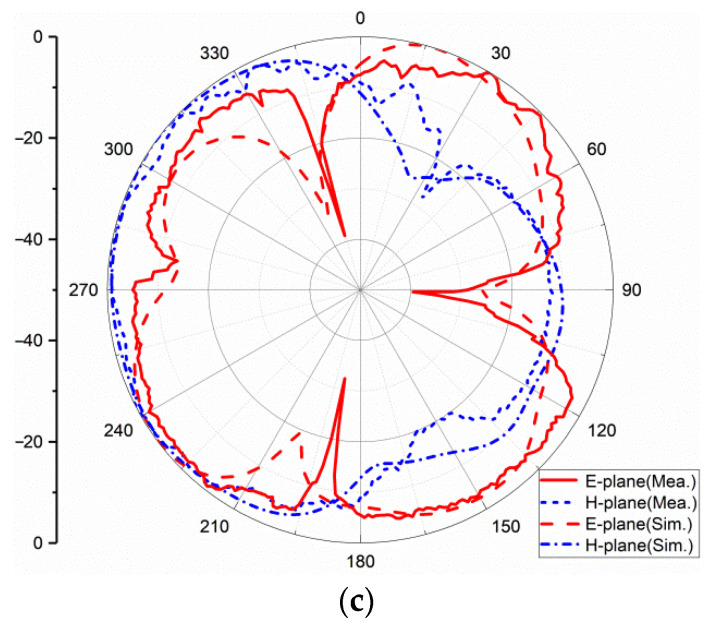
Radiation pattern at: (**a**) 3.5 GHz; (**b**) 7 GHz; (**c**) 10 GHz.

The far-field results are obtained in a microwave anechoic chamber; the excited port is connected to the testing cable and the other ports terminated to 50Ω matched loads. Figure 14 shows the normalized measured and simulated radiation patterns of one element of the antenna at 3.5 GHz, 7 GHz, and 10 GHz, respectively. The radiation pattern of the antenna is distorted with the increase in the frequency. This is mainly because the electrical size of the antenna becomes larger as the frequency increases. It is no longer an electrically small antenna, so the radiation pattern becomes distorted.

The radiation pattern is almost omni-directional in the H-plane. The 3 dB bandwidth at 3.5 GHz is 40° in the E-plane, at 7 GHz is 120° and at 10 GHz is 30°.

**Figure 15:**
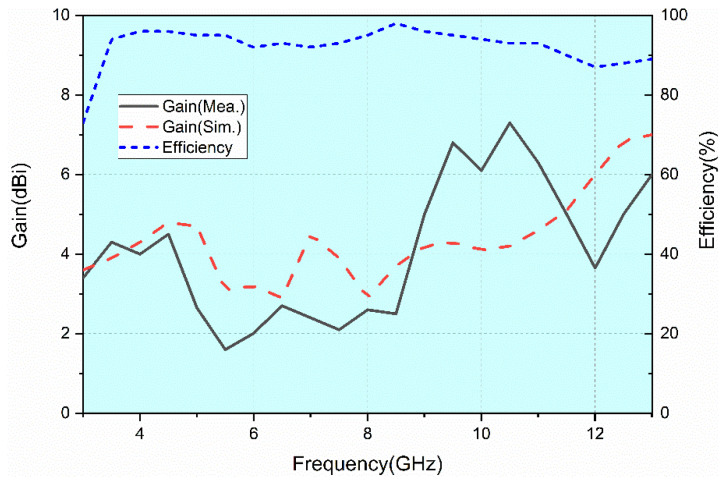
Antenna gain and radiation efficiency.

The measured gain and simulated gain are shown in Figure 15. From 3.1 GHz to 13.1 GHz, the gain value ranges from 1.6 dBi to 7.3 dBi and the average gain is 4 dBi. There are some discrepancies between the simulated and measured gain. The reason is that the SMA welded at the antenna’s ports and three 50Ω matched loads are equivalent to metal conductors, which will reflect electromagnetic waves. Therefore, these metal conductors will affect the radiation and the gain of the antenna.

The simulated radiation efficiency is shown in Figure 15. It is between 73% and 95% over the entire UWB frequency band. The radiation efficiency of the antenna is relatively stable.

The comparisons between the proposed antenna and existing ones reported in the recent publications are summarized in Table 2. With regard to the characteristics, including size, bandwidth, ECC, gain, and isolation, the proposed antenna has its own advantages compared with others.

## 4. Conclusions

A compact four-port UWB MIMO antenna with overall dimensions of 45 mm × 45 mm × 1.6 mm is described in this paper. The proposed antenna can operate in the whole UWB band (3.1–10.6 GHz) with a high isolation (−17 dB). Polarization diversity, protruded ground, and parasitic elements are used in the MIMO antenna to achieve the higher isolation. The measured return loss, isolation, and radiation pattern agree with the simulated ones. The antenna has an average gain of 4 dBi. The diversity performance of the proposed antenna is good, with an ECC less than 0.02, DG larger than 9.9985 dB, and TARC less than −25 dB. In summary, the proposed antenna is a promising candidate for UWB-MIMO wireless applications.

## Figures and Tables

**Table 1:** Geometric parameters of the proposed MIMO antenna.

Dimension	Value (mm)	Dimension	Value (mm)	Dimension	Value (mm)
W	45	Wg4	14.4	W2	1.4
L	45	Lg	10.3	W3	4.8
H	1.6	Lg1	2	Lf	11
Wg	19	Lg2	22.2	Lf1	4
Wg1	2	Lg3	2.5	Wf1	2
Wg2	0.5	Lg4	3.5	Wf2	1.3
Wg3	2.8	W1	9.5	a1	0.95
b1	15.5	a2	1.45	b2	13.5
R	7.4				

**Table 2:** Comparison of the stated UWB MIMO antennas within recent studies.

Ref.	Operating Frequency (GHz)	Bandwidth (%)	Size (λ_0_) ^1^	Gain (dBi)	ECC	Iso. (dB)	Radiation Efficiency	Substrate	No. of Elements
[2]	2.5~12	131%	0.42λ_0_ × 0.33λ_0_ (50 mm × 39.8 mm)	N/A	<0.03	>17	N/A	TMM4	4
[12]	3~11	114%	0.42λ_0_ × 0.42λ_0_ (42 mm × 42 mm)	3.5	<0.05	>15	>70%	FR4	4
[15]	3~11	114%	0.6λ_0_ × 0.6λ_0_ (60 mm × 60 mm)	3.4	<0.02	>20	>68%	FR4	4
[16]	3.1~10.6	109%	0.49λ_0_ × 0.97λ_0_ (47 mm × 93 mm)	3.5	<0.2	>31	>70%	FR4	2
[17]	3.1~17.3	139%	0.78λ_0_ × 0.78λ_0_ (75.10 mm × 75.19 mm)	5.5	<0.1	>13	N/A	FR4	4
[18]	2.7~10.6	119%	0.54λ_0_ × 0.54λ_0_ (60 mm × 60 mm)	3.5	<0.063	>15	N/A	FR4	4
[19]	3~13.2	126%	0.383λ_0_ × 0.383λ_0_ (38.3 mm × 38.3 mm)	4.1	<0.02	>17	>72%	Taconic RF-45	4
[20]	3~13.5	127%	0.58λ_0_ × 0.58λ_0_ (58 mm × 58 mm)	2.9	<0.008	>22	N/A	FR4	4
[21]	3.1~11	112%	0.46λ_0_ × 0.46λ_0_ (45 mm × 45 mm)	4.3	<0.015	>16	N/A	FR4	4
Pro.	3.1~13.1	123%	0.46λ_0_ × 0.46λ_0_ (45 mm × 45 mm)	4.0	<0.02	>17	>73%	FR4	4

^1^ λ_0_ represents the wavelength in air at the lowest frequency.

## Data Availability

Not applicable.
